# D-M159 Synergistically Induces Apoptosis in HeLa Cells Through Endoplasmic Reticulum Stress and Mitochondrial Dysfunction

**DOI:** 10.3390/ijms26073172

**Published:** 2025-03-29

**Authors:** Yuanyuan Li, Dingding Li, Zonghan Jiang, Zhihang Yuan, Zhiliang Sun, Leisheng Sun

**Affiliations:** 1 Key Laboratory of Study and Discovery of Small Targeted Molecules of Hunan Province, School of Pharmacy, Hunan Normal University, Changsha 410013, China; 18075717238@163.com (Y.L.); lidingding@hunnu.edu.cn (D.L.); 2 Hunan Engineering Research Center of Livestock and Poultry Health Care, College of Veterinary Medicine, Hunan Agricultural University, Changsha 410128, China; jiangzonghan0622@163.com (Z.J.); zhyuan2016@hunau.edu.cn (Z.Y.)

**Keywords:** peptide, D-M159, apoptosis, endoplasmic reticulum stress, mitochondrial dysfunction

## Abstract

Pore-forming peptides are promising antimicrobial and anticancer agents due to their membrane selectivity and biodegradability. Our prior work identified peptide M159, which permeabilized synthetic phosphatidylcholine liposomes without mammalian cell toxicity. Here, we report that the D-type variant (D-M159) induces apoptosis in HeLa cells under starvation. To explore its anticancer mechanism, we analyzed D-M159 cytotoxicity, intracellular uptake, and apoptotic pathways via flow cytometry, confocal microscopy, and Western blot. Calcium dynamics and mitochondrial function were examined via specific labeling and functional assays. Results revealed that D-M159 exhibited starvation-dependent, dose-responsive cytotoxicity and triggered apoptosis in HeLa cells. Mechanistic studies indicated that D-M159 entered the cells via caveolin-dependent and caveolae-dependent endocytosis pathways and induced endoplasmic reticulum stress in HeLa cells by up-regulating proteins such as ATF6, p-IRE1, PERK, GRP78, and CHOP. Meanwhile, D-M159 promoted the expression of IP3R1, GRP75, and VDAC1, which led to mitochondrial calcium iron overload, decreased mitochondrial membrane potential, and increased reactive oxygen species (ROS) generation, thereby activating the mitochondrial apoptotic pathway and inducing the aberrant expression of Bax, Bcl-2, Caspase-9, and Caspase-3. This study showed that D-M159 synergistically induced apoptosis in starved HeLa cells through endoplasmic reticulum stress and mitochondrial dysfunction, demonstrating its potential as a novel anticancer agent.

## 1. Introduction

Pore-forming peptides with novel functions have gained increasing attention due to their potential utility in many biotechnological applications, including drug delivery and antimicrobial and anticancer therapeutics [1,2]. Mechanistically, these peptides exert their killing effect on cancer cells or microbial cells by disrupting the plasma membrane and inducing cell death with selectivity [3,4,5,6]. For example, LL-37 and antimicrobial peptides from *Aspergillus* can induce both apoptosis and necrosis in tumor cells, while bee venom peptides exert antitumor effects by inhibiting tumor angiogenesis and activating immune cells [7,8,9]. Previously, we identified Melp5 as a potent and non-membrane-selective pore-forming peptide by screening a peptide library based on the sequence of bee venom, melittin [10,11]. Subsequently, to increase the membrane selectivity of the peptide, we discovered peptide M159 by screening a second-generation peptide library using Melp5 as a template [12]. M159 is highly membrane selective, with the most potent permeabilizing activity in pure phosphatidylcholine (PC) bilayers. Unlike Melp5, M159 is much less capable of permeabilizing synthetic membranes containing cholesterol [11]. While melittin and Melp5 are highly cytotoxic, macrolittins have no measurable lytic activity against mammalian cells even at high peptide concentrations (Table 1). Interestingly, we found that D-type M159 (D-M159) instead of L-type M159 (M159) induced cell apoptosis under starvation conditions. However, the potential mechanisms underlying D-M159-induced apoptosis need to be elucidated.

The endoplasmic reticulum (ER) and mitochondria are crucial for maintaining cellular homeostasis, protein synthesis, lipid metabolism, and calcium ion balance and for responding to intracellular stresses [13,14]. For example, ER stress activates key molecules, including ATF6, p-IRE1, PERK, CHOP, and Grp78, which then trigger apoptosis [15]. Mitochondrial dysfunction activates Caspase-9 and Caspase-3, which regulate the balance between Bax and Bcl-2, ultimately leading to apoptosis [16]. As cancer biology research advances, alterations in the endoplasmic reticulum and mitochondrial function have been found to be closely linked to key biological processes, including the proliferation, survival, migration, and invasion of cancer cells [17]. It has been reported that the bovine lactoferrin B polypeptide has been shown to induce apoptosis in human leukemia and cancer cell lines via the mitochondrial pathway, primarily through the generation of reactive oxygen species [18]. The cationic antimicrobial peptide NRC-03 induces apoptosis in oral squamous carcinoma cells through mitochondrial oxidative stress mediated by the CypD-mPTP axis [19]. Furthermore, it has been shown that the IP3R1-GRP75-VDAC1 complex acts as a bridge between the endoplasmic reticulum and mitochondria by regulating calcium ions released from the endoplasmic reticulum, which in turn would affect mitochondrial function [20].

In this work, we examined the effects of D-M159 on ER function, mitochondrial function, the IP3R1-GRP75-VDAC1 complex, and cell lysis. We found that D-M159 combined with starvation treatment induced endoplasmic reticulum dysfunction, mitochondrial oxidative stress, and ultimately apoptosis in HeLa cells by modulating the IP3R1-GRP75-VDAC1 complex. This suggests that D-M159 may be a promising candidate for combination with starvation treatment to enhance its anticancer effect, overcome the challenges of the tumor microenvironment, and improve therapeutic efficacy, positioning it as a potential new cancer therapeutic agent.

## 2. Results

### 2.1. D-M159 Combined Starvation Treatment Induces Cell Death

**Figure 1:**
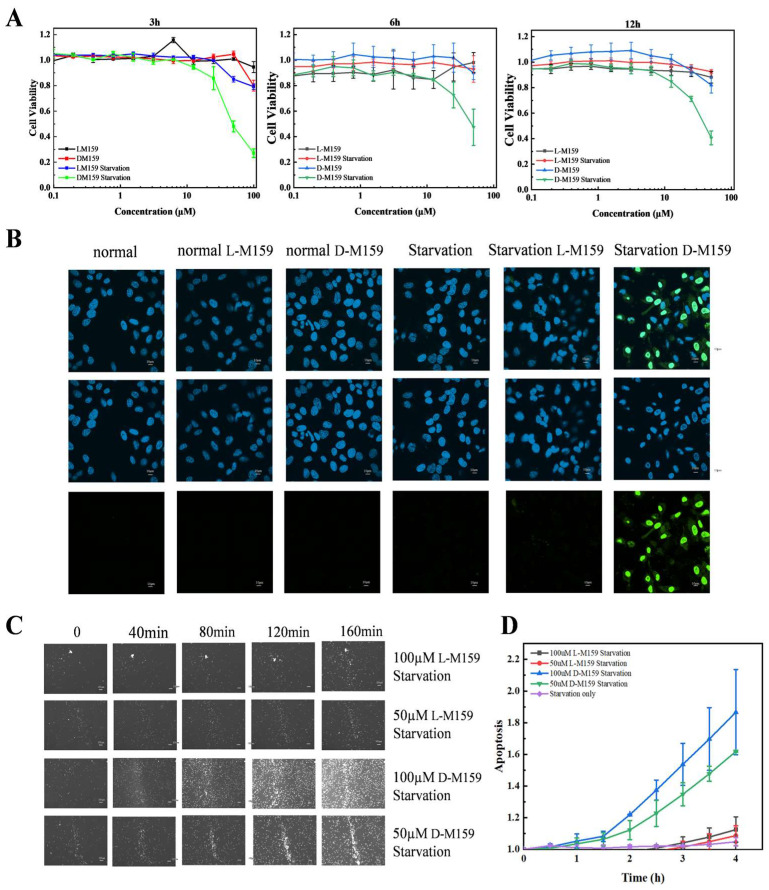
D-M159 combined with starvation treatment induces cell death. (**A**) Alamar blue cytotoxicity assay. (**B**) Immunofluorescence of caspase-3 activity (Scale = 10 μm). (**C**,**D**) Time course tracking technique to detect caspase-3 activity (Scale = 100 μm). For (**A**) (*n* = 3) and (**D**) (*n* = 3), values are expressed as mean ± SD.

Cytotoxicity of both types of M159 was evaluated under normal and nutrient-deprived conditions. The results showed that D-M159 was effective in inducing cell death in HeLa cells under starvation conditions in a dose-dependent manner in the range of 10 µM to 100 µM. In contrast, L-M159 treatment caused no significant cellular changes, consistent with our previous findings (Figure 1A). Caspase-3 is upregulated during apoptosis-induced cell death. The activity of Caspase-3 in HeLa cells was assessed, and no significant green fluorescence was observed under normal conditions following treatment with L-M159 and D-M159. However, under starvation conditions, the intensity of cellular fluorescence increased after L-M159 treatment, with a stronger fluorescence observed following D-M159 treatment compared to L-M159. These results suggest that D-M159 significantly activates Caspase-3 and induces cell death under starvation conditions (Figure 1B). Based on these findings, we focused on the effects of L-M159 and D-M159 on HeLa cells under starvation conditions. The dynamic analysis of the death process was achieved by detecting the activation of caspase-3 at specific time points by the time-tracking technique. The results further confirmed D-M159 treatment under starvation conditions in a time-dependent manner (Figure 1C,D).

### 2.2. D-M159 Enters HeLa Cells by Specific Endocytosis

**Figure 2:**
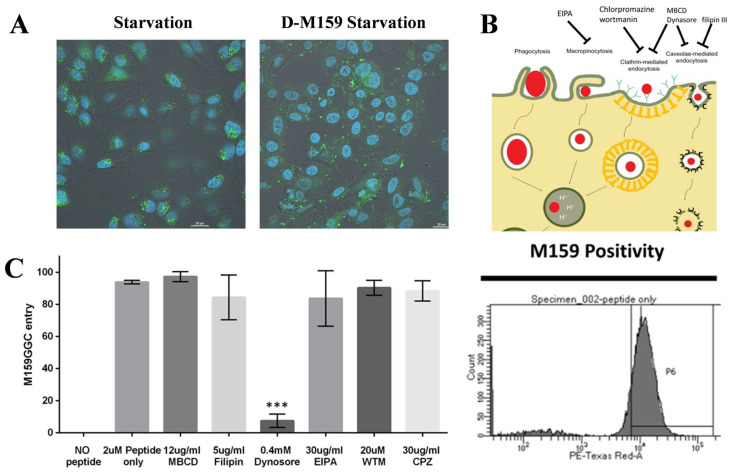
D-M159 specifically enters the cytoplasm of HeLa cells. (**A**) D-M159 facilitates the transport of 10 kDa dextran (Scale = 20 μm). (**B**) Schematic representation of various inhibitors of the endocytosis pathway. (**C**) The pathways of D-M159 entry into cells were determined using various inhibitors; for (**C**) (*n* = 3), values are expressed as mean ± SD. Compared with blank, *** *p* < 0.001.

Dextran is a complex and heterogeneous polysaccharide that can be synthesized at many different high molecular weights. HeLa cells were co-incubated under starvation conditions using 10 kDa Dextran with 100 μM D-M159. It was observed that the fluorescence intensity within HeLa cells was enhanced in the D-M159-treated group compared to the starved group, suggesting that D-M159 treatment enhances the permeability of HeLa cells (Figure 2A). There are four main classes of humoral uptake pathways: macropinocytosis, clathrin-mediated, caveolae-mediated, and clathrin- and caveolae-independent pathways. Next, we tested the first three pathways by using multiple inhibitors to determine which of these pathways dominate D-M159 uptake. Methyl-β-cyclodextrin (MBCD) inhibits the formation of caveolae and clathrin vesicles by depleting cholesterol from the cell membrane. Chlorpromazine (CPZ), as an inhibitor of the clathrin adaptor protein AP2, effectively blocks clathrin-mediated endocytosis. Filipin disrupts caveolar structures and impedes their proper assembly and function. Wortmannin inhibits PI3-kinase, thereby disrupting clathrin-mediated endocytosis. Dynasore inhibits dynamin, a key player in clathrin vesicle scission from the plasma membrane, and also interferes with caveolae dynamics due to its interaction with Cav1. Finally, EIPA blocks sodium channels, thereby inhibiting macropinocytosis (Figure 2B). The results revealed that only Dynasore, an inhibitor that affects clathrin-mediated and caveolae-mediated endocytosis, could block D-M159 entry into HeLa cells (Figure 2C).

### 2.3. Transcriptome Analysis of HeLa After Exposure to D-MI59 and Starvation

**Figure 3:**
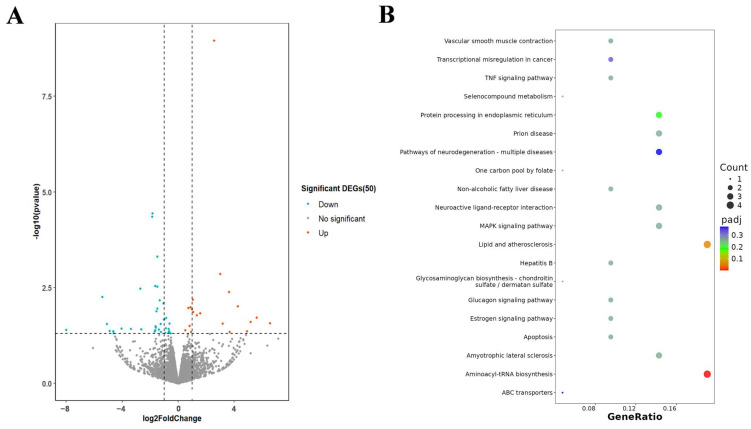
Transcriptome analysis of HeLa after exposure to D-MI59 and starvation. (**A**) Volcano plots of DEGs. (**B**) KEGG functional analysis of DEGs.

To further investigate the potential mechanism of the pro-apoptotic effect of D-M159 combined with starvation treatment, the researchers extracted total RNA from HeLa cells of treated and untreated groups for RNAseq analysis. Transcriptomics analysis revealed the presence of DEGs between the control and treated groups (Figure 3A). KEGG enrichment analysis revealed that D-M159 has a significant effect on signaling in HeLa cells, including apoptosis, protein processing in the endoplasmic reticulum, and via the MAPK signaling pathway and TNF signaling pathway (Figure 3B). These signaling cascades have been shown to be involved in the apoptotic process.

### 2.4. D-M159 Combined Starvation Treatment Induces Cell Apoptosis

**Figure 4:**
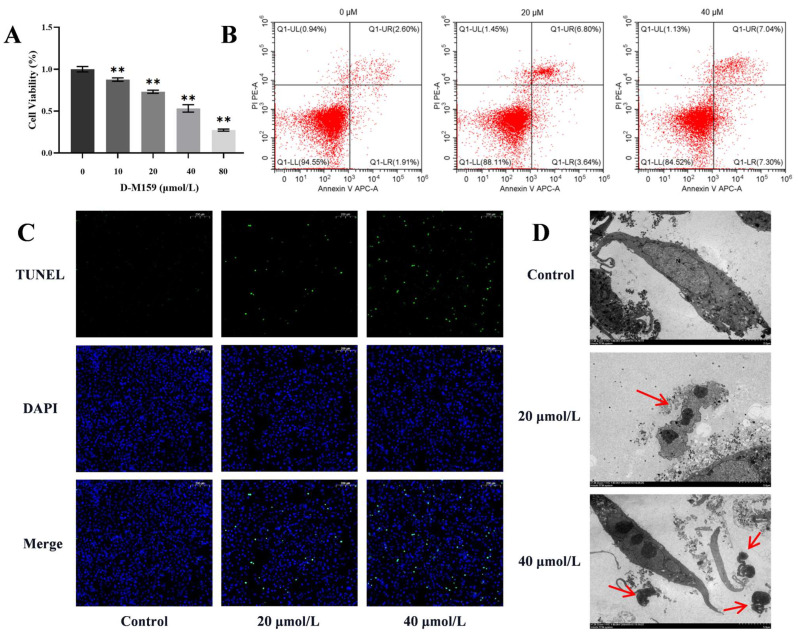
D-M159 combined with starvation treatment induces apoptosis. (**A**) CCK8 cytotoxicity assay. (**B**) Flow cytometry detection of apoptosis. (**C**) TUNEL staining. (Scale = 200 μm) (**D**) TEM observation images with red arrows pointing to apoptotic vesicles. (Scale = 5 μm) For (A) (*n* = 3), values are expressed as mean ± SD. Compared with blank, ** *p* < 0.01.

To confirm the cytotoxicity of D-M159, CCK-8 assays were conducted, and we found that D-M159 induced HeLa cell death in a dose-dependent manner, with 50% cell death occurring at 40 µM, while a significant reduction in cell death was observed at 20 µM. Therefore, these two concentrations were selected for subsequent experiments (Figure 4A). The annexin/propidium iodide (PI) staining assay was used to detect changes in cell membrane permeability and apoptosis. Flow cytometry revealed a dose-dependent increase in HeLa cell apoptosis following D-M159 treatment combined with starvation (Figure 4B). Additionally, TUNEL staining showed that fluorescence intensity was positively correlated with the D-M159 dose (Figure 4C), further confirming that D-M159 promotes HeLa cell apoptosis. Transmission electron microscopy (TEM) analysis also revealed an increase in apoptotic vesicles in HeLa cells following D-M159 treatment (Figure 4D). In conclusion, these findings validate that D-M159 combined with starvation induces apoptosis in HeLa cells.

### 2.5. D-M159 Combined with Starvation Treatment Induces Endoplasmic Reticulum Stress in HeLa Cells

**Figure 5:**
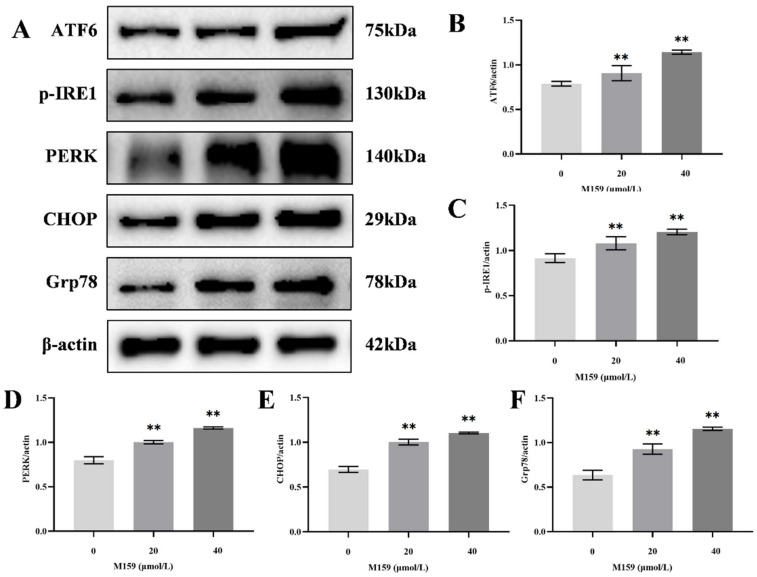
D-M159 combined with starvation treatment induced apoptosis of the endoplasmic reticulum pathway in HeLa cells. (**A**–**F**) Western blot detection of the expression of endoplasmic reticulum functional proteins ATF6, p-IRE, PERK, GRP78, and CHOP; for (**A**–**F**) (*n* = 3), values are expressed as mean ± SD. Compared with blank, ** *p* < 0.01.

The endoplasmic reticulum (ER) is a crucial organelle for maintaining cellular homeostasis. Under stressful conditions, the expression of key proteins involved in the three major pathways of ER stress, ATF6, p-IRE1, and PERK, is upregulated. CHOP and Grp78 are marker proteins downstream of these pathways. To assess ER stress, we examined the expression of these proteins by Western blot. The results demonstrated a significant increase in the protein levels of ATF6, p-IRE1, PERK, CHOP, and Grp78 in the 20 µM and 40 µM D-M159 groups compared to the control group (Figure 5A–F). These findings suggest that D-M159 induces ER stress in HeLa cells.

### 2.6. D-M159 Combined with Starvation Treatment Disrupts Intracellular Calcium Homeostasis in HeLa Cells

**Figure 6:**
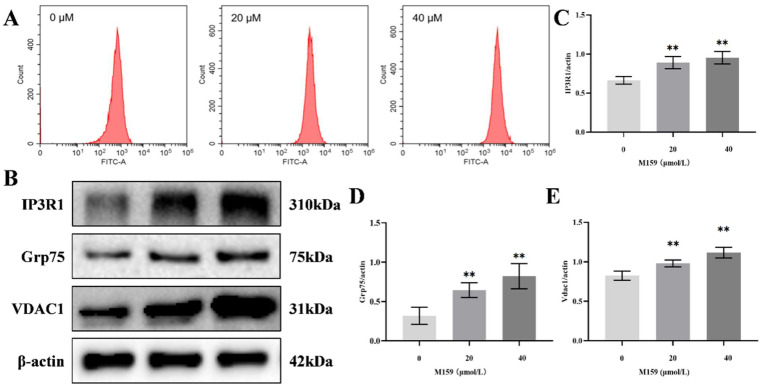
D-M159 combined with starvation treatment disrupts intracellular calcium ion homeostasis in HeLa cells. (**A**) Intracellular calcium ion levels. (**B**–**E**) Western blot detection of protein expression of IP3R1, GRP75, and VDAC1. For (**B**–**E**) (*n* = 3), values are expressed as mean ± SD. Compared with blank, ** *p* < 0.01.

Intracellular calcium ion content plays a crucial role in regulating apoptosis, with excessive calcium ion accumulation activating the mitochondrial pathway of apoptosis. Flow cytometry analysis of intracellular calcium ions in HeLa cells revealed an increase in calcium levels following treatment with 20 µM and 40 µM D-M159 compared to the control group (Figure 6A). IP3R1, GRP75, and VDAC1 are protein channels that facilitate the transport of calcium ions from the endoplasmic reticulum to the mitochondria. Consequently, Western blot analysis showed an abnormal upregulation of IP3R1, GRP75, and VDAC1 in the 20 µM and 40 µM D-M159-treated groups compared to the control group (Figure 6B–E). These findings suggest that calcium ion delivery to the mitochondria is enhanced, leading to a disruption of calcium homeostasis.

### 2.7. D-M159 Co-Starvation Treatment Induces Mitochondrial Pathway Apoptosis in HeLa Cells

**Figure 7:**
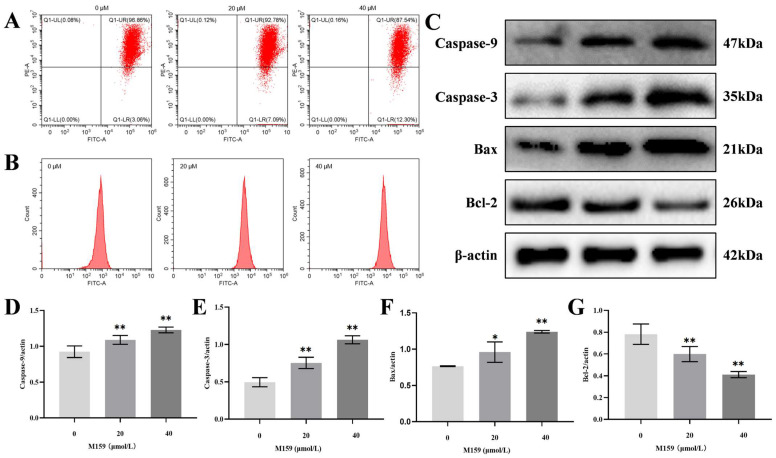
D-M159 combined with starvation treatment induces apoptosis by the mitochondrial pathway in HeLa cells. (**A**) Mitochondrial membrane potential levels. (**B**) Intracellular ROS levels. (**C**–**G**) Protein expression of Caspase-9, Caspase-3, Bax, and Bcl-2 was detected by Western blot. For (**C**–**G**) (*n* = 3), values are expressed as mean ± SD. Compared with blank, * *p* < 0.05, ** *p* < 0.01.

Increased calcium ion accumulation in the mitochondria leads to mitochondrial dysfunction. Mitochondrial membrane potential (MMP) decreases during the initiation of apoptosis. In this study, changes in the mitochondrial membrane potential of HeLa cells treated with D-M159 combined with starvation were assessed using flow cytometry. A dose-dependent decrease in mitochondrial membrane potential was observed in HeLa cells treated with 20 µM and 40 µM D-M159 compared to the control group (Figure 7A). Mitochondria are both a major source of reactive oxygen species (ROS) and a primary target of ROS-induced cellular damage. Flow cytometry analysis revealed a dose-dependent increase in intracellular ROS levels in HeLa cells treated with 20 µM and 40 µM D-M159 compared to the control group (Figure 7B). In addition, our Western blot results showed that the protein expression levels of Caspase-9 and Caspase-3 were significantly increased in the 20 µM and 40 µM D-M159-treated groups compared to the control group. Moreover, the expression of the anti-apoptotic protein Bcl-2 was significantly decreased, while the expression of the pro-apoptotic protein Bax was significantly increased in the 20 µM and 40 µM D-M159-treated groups. These findings further suggest that D-M159, in combination with starvation, induces apoptosis in HeLa cells through the mitochondrial pathway (Figure 7C–G).

## 3. Discussion

Peptides are a class of small-molecule compounds composed of amino acids, which show a wide range of applications in the fields of anticancer, anti-infection, and anti-inflammation. Compared with traditional drugs, peptide drugs have gradually become a hotspot for drug development in recent years due to their advantages of high specificity, low toxicity, and easy metabolic degradation [21]. For example, the clinically applied peptide drug bortezomib has demonstrated good activity and tolerability in patients with relapsed multiple myeloma with varying degrees of renal insufficiency [22]. A peptide used in the treatment of breast cancer has been found to bind to the HER2 receptor of cancer cells. This peptide was then converted into nanofibrils, disrupting HER2 dimerization and its downstream signaling and further inducing apoptosis of cancer cells while suppressing the expression of proliferation- and survival-related genes [23]. In the present study, we initially found that the novel peptide D-M159 demonstrated a significant role in inducing cell death. Specifically, our results showed that D-M159 was able to significantly induce apoptosis in HeLa cells under starvation conditions, whereas its isomer L-M159 did not exhibit similar effects in either starved or non-starved states. This suggests that D-M159 has a unique cytotoxic effect. Endocytosis pathways are divided into four main categories: macropinocytosis, the lattice protein-mediated pathway, the vesicular protein-mediated pathway, and pathways independent of lattice proteins and vesicular proteins [24]. Therefore, we found that D-M159 uptake relies on caveolin-dependent and caveolae-dependent endocytosis mechanisms by probing with multiple inhibitors in the cell. Caspase-3 has been reported to be the execution protein of apoptosis, and its activity is regulated by initiating caspases, including Caspase-9 and Caspase-12 [25]. Our study showed that D-M159 combined with starvation treatment significantly up-regulated the protein expression of Caspase-3 and enhanced the fluorescence signals of Caspase-3 and TUNEL fluorescence signaling, further validating its ability to induce apoptosis. By the CCK-8 assay and flow cytometry analysis, we further confirmed that D-M159 combined with starvation treatment caused a significant dose-dependent increase in apoptosis in HeLa cells. This effect was similar to the mechanism by which the anticancer peptide pClusters effectively inhibits tumor growth and metastasis in multiple animal models by impairing the Wnt/β-catenin pathway [26].

Apoptosis is a tightly regulated form of programmed cell death that plays a key role in the maintenance of tissue homeostasis and removal of damaged cells, and it is closely related to mitochondrial function, calcium homeostasis, and endoplasmic reticulum stress. Mitochondria-mediated apoptosis is of great significance in cancer research because its dysregulation is often a key reason why cancer cells escape death. Cancer cells that initiate mitochondrial apoptosis have been reported to be more sensitive to NK cell-mediated killing [27]. In the present study, D-M159 was found to exert a significant anticancer effect by inducing apoptosis in the mitochondrial pathway of HeLa cells. It was shown that the expression of the pro-apoptotic protein Bax was significantly up-regulated and the expression of the anti-apoptotic protein Bcl-2 was down-regulated after D-M159 treatment. This mechanism of action is similar to that of Alisol B 23-acetate, which induces apoptosis in ovarian cancer cells by regulating the Bax/Bcl-2 ratio [28]. In addition, D-M159 increases the expression of IP3R1, GRP75, and VDAC1 proteins, which enhances endoplasmic reticulum-to-mitochondria calcium ion transfer, further exacerbating mitochondrial dysfunction [29]. This mechanism of action is consistent with the findings for other peptide drugs. It has been reported that sIL-24 induces apoptosis in the mitochondrial pathway by increasing the protein ratio of Bax/Bcl-2, increasing cytochrome C release, and up-regulating the expression of Caspase-9 and Caspase-3 in cancer cells [30]. This suggests that D-M159 is capable of inducing the expression levels of Bax, Bcl-2, Caspase-9, and Caspase-3 abnormally, thereby disrupting calcium homeostasis and initiating the mitochondria-mediated apoptotic pathway.

Mitochondrial calcium ion overload is an important inducer of apoptosis. Excessive calcium ions in the mitochondria trigger the generation of reactive oxygen species (ROS), the decrease of mitochondrial membrane potential, and the activation of mitochondria-associated apoptotic proteins [31]. In the present study, we showed that D-M159 combined with starvation treatment significantly induced the occurrence of mitochondrial calcium ion overload in HeLa cells, which led to the increase of ROS generation and triggered the apoptotic process through the down-regulation of the mitochondrial membrane potential. Notably, the endoplasmic reticulum, as an important site for protein synthesis and folding, also plays a key role in maintaining cellular homeostasis and transmitting death signals [32]. The results showed that D-M159 induced HeLa cells to undergo endoplasmic reticulum stress by significantly up-regulating the expression of ATF6, p-IRE1, PERK, CHOP, and Grp78 proteins, which further promoted apoptosis. This mechanism is similar to that reported in the literature for the induction of apoptosis in colon cancer cells by andrographolide through activation of endoplasmic reticulum stress [33]. In addition, activation of CHOP inhibits the expression of the pre-apoptotic protein Bcl-2, which initiates the mitochondria-mediated apoptotic pathway [34].

Transcriptomic data suggest that D-M159 synergistically regulates ERS and mitochondrial dysfunction through activation of the MAPK and TNF pathways. Specifically, phosphorylation of the MAPK pathway (e.g., p38/JNK) promotes ATF4/CHOP axis expression and enhances terminal apoptotic signaling in ERS [35], which is consistent with the up-regulation of CHOP and the Bax/Bcl-2 ratio observed in this study. Meanwhile, activation of the TNF-α/NF-κB pathway may weaken mitochondrial homeostasis through a dual mechanism: on the one hand, NF-κB inhibits the Bcl-2 family of anti-apoptotic proteins (e.g., Bcl-xL), which directly disrupts the mitochondrial membrane integrity [36]; on the other hand, TNF-α-induced ROS bursts can damage the mitochondrial electron transport chain, resulting in potential membrane collapse, further amplifying apoptotic signaling [37]. Notably, there may be cross-regulation between MAPK and TNF pathways: for example, TNF-α-activated JNK phosphorylates Bcl-2, contributing to its dissociation from mitochondria and loss of function [38], whereas sustained activation of NF-κB may inhibit the IRE1α-XBP1 pathway and aggravate the apoptotic tendency of ERS [39]. This multi-pathway synergy reveals the molecular logic of D-M159’s potent pro-apoptotic effect through the ERS–mitochondria–inflammation signaling network, providing a theoretical basis for targeted multi-node intervention.

Thus, D-M159 synergistically promotes apoptosis in HeLa cells by inducing endoplasmic reticulum stress and mitochondrial dysfunction, which provides a new way of exploring the anticancer mechanism of the peptide and demonstrates its potential value in anticancer therapy.

## 4. Materials and Methods

### 4.1. Reagents

M159 was purchased from GL Biochem (Shanghai) Ltd., China, and the peptide sequence was GIGEVLHELATLLPELWIKAAQQL. All peptides were prepared by solid-phase synthesis with purity > 99.5%. Methyl-β-cyclodextrin (MBCD), Chlorpromazine (CPZ), Filipin, Wortmannin, Dynasore, EIPA, and 10 kDa dextran-AF488 were purchased from MCE, (Monmouth Junction, NJ) Ltd., USA. ATF6, p-IRE1, PERK, IP3R1, GRP75, VDAC1, and β-actin used in protein immunoblotting experiments were purchased from Abclone (Wuhan) Ltd., China. GRP78, CHOP, Caspase-9, Caspase-3, Bax, and Bcl-2 were purchased from WanLei (Shengyang) Ltd., China. Cell serum was purchased from TransGen (Beijing) Ltd., China, and a 1% penicillin-streptomycin solution, Alamar Blue reagent, DEVD-NucView 488, flow cytometry kit, and TUNEL staining kit were purchased from MCE, (New Jersey) Ltd., USA. DMEM with high glucose was purchased from MCE, (New Jersey) Ltd., USA. The Cell Counting Kit-8 was purchased from YEASEN (Shanghai) Ltd., China. The RNeasy mini kit was purchased from Qiagen, Hilden, Germany. The TruSeq RNA Sample Preparation Kit was purchased from Illumina, San Diego, CA, USA.

### 4.2. Cell Culture

Human cervical cancer cells (HeLa) were cultured in DMEM with high glucose containing 10% fetal bovine serum and a 1% penicillin-streptomycin solution. Cells were maintained in a humidified incubator at 37 °C with 5% CO_2_. The trypsin solution was used for cell passaging, and cells in good condition after three generations were selected for experiments. The cells were treated with the EBSS buffer or L/D-M159.

### 4.3. Alamar Blue Cytotoxicity Assay

The Alamar Blue assay was performed to assess cell viability and cytotoxicity. After the treatment of HeLa cells with various concentrations of the test compound (M-159 or other agents), cells were incubated for 24, 48, or 72 h. Following treatment, the medium was replaced with 100 µL of a fresh culture medium containing a 10% Alamar Blue reagent and incubated for 4 h at 37 °C. The fluorescence intensity was measured using a microplate reader at excitation and emission wavelengths of 540 nm and 590 nm, respectively.

### 4.4. CCK-8 Cell Viability Assay

To assess cell viability and cytotoxicity, HeLa cells were seeded in 96-well plates at a density of 1 × 10^4^ cells per well in 100 µL of a culture medium and incubated for 24 h. After the cells adhered, the culture medium was replaced with a fresh medium containing varying concentrations of the experimental compounds. Cells were then incubated for an additional 3 h, depending on the experimental design. After incubation, 10 µL of the Cell Counting Kit-8 (CCK-8) reagent was added to each well, and the cells were further incubated for 1 h at 37 °C. The absorbance was measured at 450 nm using a microplate reader.

### 4.5. Caspase-3 Activity Assay

After HeLa cells were treated with the appropriate reagents, the Caspase-3 activity assay was performed according to the kit instructions. Briefly, treated cells and the DEVD-NucView 488 (Caspase-3 substrate) were co-incubated, and Caspase-3 activity was monitored in real time using fluorescence confocal microscopy. Cell nuclei were subsequently labeled with DAPI and imaged by fluorescence microscopy.

### 4.6. Detection of Apoptosis by Flow Cytometry

HeLa cells were treated with the appropriate reagents, digested with trypsin, washed with PBS, centrifuged, resuspended in a binding buffer, and stained with Annexin V-FITC and PI according to the prescribed procedure. Cells were incubated at room temperature for 15 min protected from light. Finally, the percentage of apoptotic and necrotic cells was assessed by flow cytometry analysis.

### 4.7. TUNEL Staining for Apoptosis Detection

HeLa cells were fixed with 4% paraformaldehyde for 10–15 min at room temperature after treatment with the appropriate reagents, permeabilized with 0.1% Triton X-100 (MCE, New Jersey, USA) followed by the addition of the TUNEL reaction solution, and incubated at 37 °C for 1 h with light protection. At the same time, the nuclei were stained with DAPI and imaged by fluorescence microscopy.

### 4.8. Transmission Electron Microscopy (TEM)

HeLa cells were fixed in a 2.5% glutaraldehyde solution for 12 h. The samples were washed three times with phosphate-buffered saline, then post-fixed in 1% osmium tetroxide for 2 h. After gradient dehydration, the samples were embedded in pure acetone and an embedding medium. The blocks were polymerized in an oven. Ultrathin sections (50–60 nm thick) were cut using an ultramicrotome, stained, and imaged using a transmission electron microscope.

### 4.9. Dextran Delivery

For Dextran delivery, HeLa cells were seeded at a density of 1 × 10^5^ cells per well in 24-well plates and allowed to adhere overnight. Cells were then treated with 100 µg/mL 10 kDa dextran-AF488 in serum-free EBSS for 2 h. Following treatment, the medium was aspirated, and cells were washed twice with PBS to remove any uninternalized Dextran. The cells were then fixed with 4% PFA for 10 min at room temperature. After fixation, the cells were permeabilized with 0.1% Triton X-100 for 10 min and washed three times with PBS. To visualize Dextran uptake, cell nuclei were stained with DAPI (1 μg/mL) for 5 min. Fluorescence microscopy was used to observe the intracellular uptake of FITC-labeled Dextran.

### 4.10. D-M159-TAMRA Conjugation

TAMRA was conjugated to D-M159-GC using a maleimide–thiol reaction. Appropriate amounts of D-M159-GC and TAMRA-maleimide were weighed out and dissolved together to make 1.5 mM D-M159-GC and 6 mM TAMRA maleimide in a degassed 10 mM phosphate buffer. Some maleimide crystals remained. The reaction was tracked with HPLC over 3 h at room temperature, at which time the reaction was deemed complete. The conjugate was then purified with HPLC, and the molecular weight was verified with mass spectrometry.

### 4.11. Endosomal Pathway Determination Using Inhibitors

HeLa cells were plated on a 48-well plate overnight at 37 °C 5% CO_2_. Cells were washed with PBS and inhibitor was added, 30 min at 37 °C. Optimal concentrations of the inhibitor were determined previously by using a sub-cytotoxic concentration as determined by Alamar blue. Concentrations were as follows: 12 µg/mL methyl-β-cyclodextrin (MBCD), 5 µg/mL filipin III, 30 µg/mL 5-(N-ethyl-N-isopropyl) amiloride (EIPA), 20 µM wortmannin, 0.4 mM dynasore, and 30 µg/mL chlorpromazine. D-M159-TAMRA was then added directly to the wells and incubated for 45 min at 37 °C. Cells were washed in PBS and then trypsinized. Complete media was then added, transferred to a 1.5 mL tube, spun down, then resuspended in eBioscience viability dye e780 for 10 min at room temperature. Cells were washed, spun down, then resuspended in FACS buffer before performing flow cytometry.

### 4.12. RNA Sequencing

The total RNA of HeLa cells treated for 4 h with or without D-M159 and starvation was isolated using an RNeasy mini kit. Paired-end libraries were synthesized by using the TruSeq RNA Sample Preparation Kit following the TruSeq RNA Sample Preparation Guide. Purified libraries were quantified by a Qubit 2.0 Fluorometer (Thermo Fisher Scientific, Waltham, MA, USA) and validated by an Agilent 2100 bioanalyzer (Agilent Technologies, Santa Clara, CA, USA) to confirm the insert size and calculate the mole concentration. A cluster was generated by cBot, with the library diluted to 10 pM and then sequenced on the Illumina NovaSeq 6000 by Abiowell Biotechnology Co., Ltd., Changsha, China. The reads were aligned with Hisat2 (v 2.1.0) to GRCm38 with default parameters. The output SAM (sequencing alignment/map) files were converted to BAM (binary alignment/map) files and sorted using SAMtools (version 1.3.1). Gene abundance was expressed as fragments per kilobase of exon per million reads mapped (FPKM). StringTie software (https://github.com/gpertea/stringtie) was used to count the fragment within each gene, and the TMM algorithm was used for normalization35. Differential expression analysis for mRNA was performed using R package edgeR (https://bioconductor.org/packages/edgeR). Differentially expressed RNAs with a |log_2_(FC)| value > 1 and q value < 0.05, considered to be significantly modulated, were retained for further analysis.

### 4.13. Western Blot Analysis

Proteins were separated on a 6–15% SDS-PAGE gel and transferred to a PVDF membrane. After being closed with 5% skimmed milk for 2 h, the membrane was incubated with the primary antibody at 4 °C overnight. After a TBST wash, the membrane was incubated with the secondary antibody for 1 h. After washing again, the membrane was visualized using the ECL reagent purchased from YEASEN, and protein bands were analyzed by Image Lab software (6.1.0 WIN).

### 4.14. ROS Analysis

HeLa cells were seeded into 6-well plates at a density of 1 × 10^5^ cells per well and allowed to adhere overnight. The cells were then treated with the experimental compounds for the desired time points. After treatment, cells were washed twice with PBS and incubated with 10 μM DCFH-DA for 30 min at 37 °C, protected from light. Following incubation, cells were washed twice with PBS to remove excess DCFH-DA and resuspended in 500 μL of PBS for analysis. ROS levels were assessed using flow cytometry, and the fluorescence intensity was measured in the FITC channel (emission at 530 nm and excitation at 485 nm).

### 4.15. Mitochondrial Membrane Potential (ΔΨm) Measurement

Mitochondrial membrane potential (MMP) was measured using the Mitochondrial Membrane Potential Assay Kit (MCE, New Jersey, USA). Briefly, HeLa cells were treated with the experimental conditions for 24 h. After treatment, cells were rinsed twice with PBS and then incubated with the JC-1 staining solution at a final concentration of 10 μM for 20 min at 37 °C in a dark environment. At the end of the incubation, cells were rinsed twice with PBS to remove excess dye. To determine the mitochondrial membrane potential, the cells were analyzed by flow cytometry, and the JC-1 dye fluoresced red (590 nm) when aggregated in healthy mitochondria and green (530 nm) when monomers were present under depolarizing conditions.

### 4.16. Statistical Analysis

Data from independent triplicate experiments were analyzed using GraphPad Prism 8.2 for graphical representation and SPSS 23.0 for statistical analysis. Results are expressed as “mean ± standard deviation (X ± SD)”. A *p*-value of <0.05 was considered statistically significant, and *p* < 0.01 was considered highly significant. One-way analysis of variance (ANOVA) was used for comparison analysis.

## 5. Conclusions

In summary, the present study systematically revealed the mechanism of action of the novel peptide D-M159 combined with starvation treatment in HeLa cells. D-M159 promoted apoptosis in HeLa cells by significantly inducing endoplasmic reticulum stress and mitochondrial dysfunction. These findings not only provide an important experimental basis for the development of D-M159 as a potential anticancer drug but also open up a new direction for the exploration of the application of peptide drugs in tumor therapy. Future studies will focus on optimizing the molecular structure of D-M159 and its pharmacokinetic properties to further enhance its efficacy and safety and lay a solid foundation for its clinical translation.

## Figures and Tables

**Table 1:** Melittin and its derived peptides.

Pore-Forming Peptide	Sequence	IC_50_ (μM)	Permeability to PC Bilayers
Melittin	GIGAVLKVLTTGLPALISWIKRKRQQ	1–5	low
Melp5	GIGAVLKVLATGLPALISWIKAAQQL	1–5	medium
M159	GIGEVLHELATLLPELISWIKAAQQL	>200	high

## Data Availability

The original contributions presented in this study are included in the article. Further inquiries can be directed to the corresponding authors.
